# Muscular Strength and Quality of Life in Older Adults: The Role of *ACTN3 R577X* Polymorphism

**DOI:** 10.3390/ijerph18031055

**Published:** 2021-01-25

**Authors:** Ana Fernández-Araque, Andrea Giaquinta-Aranda, Jose Andrés Rodríguez-Díez, Silvia Carretero-Molinero, Jorge López-López, Zoraida Verde

**Affiliations:** 1 Department of Nursery, Campus Duques de Soria, Universidad de Valladolid, 42004 Soria, Spain; anamaria.fernandez@uva.es (A.F.-A.); agaranda1993@hotmail.com (A.G.-A.); silvia.carretero@uva.es (S.C.-M.); 2 Department of Biochemistry, Molecular Biology and Physiology, Campus Duques de Soria, Universidad de Valladolid, 42004 Soria, Spain; joseandres.rodriguez@uva.es (J.A.R.-D.); jorgelopez81@gmail.com (J.L.-L.)

**Keywords:** ACTN3, genetic variant, elderly population, quality of life, mobility, hand grip strength, multimorbidity

## Abstract

As longevity is increasing, the 65-year-old and older population is projected to increase in the next decades, as are the consequences of age-related muscle deterioration on the quality of life. The purpose of this study was to examine the associations of the *ACTN3* *R577X* polymorphism with quality of life and muscular strength in an older Spanish population. In total, 281 older adults participated in this study. Anthropometric measurements, chronic diseases, prescribed medications, quality of life, hand grip strength, and physical activity and nutritional status data were collected. *ACTN3 R577X* genotyping was determined using Taqman probes. Multivariate regression analysis revealed in adjusted model that, in men, the *ACTN3 R577X* genotype was significantly associated with hand grip strength (HGS), regression coefficient (β) = 1.23, *p* = 0.008, dimension 1 of the five-dimension questionnaire EuroQoL (EQ-5D, mobility), (β) = −1.44, *p* = 0.006, and clinical group risk (CGR) category (β) = −1.38, *p* = 0.006. In women, a marginal association between the *ACTN3 R577X* genotype and the CGR category was observed, with a regression coefficient of (β) = −0.97, (*p* = 0.024). Our findings suggest that the *ACTN3 R577X* genotype may influence the decline in muscle strength and quality of life in older Spanish adult males.

## 1. Introduction

Ageing increases the levels of functional dependency of older adults, which has individual and social implications. Therefore, a new way of thinking about and measuring aging in a socioeconomic and sanitary context is necessary [1]. Societies have to improve the robustness of assistance systems in Europe and encourage older adults to be active and healthy [2].

In recent studies, physical activity (PA) has appeared to be associated with better physical health and has thus become a priority of public health systems for a better quality of ageing [3,4]. Successful ageing is a multidimensional concept defined as good physical, psychological, and social functioning in old age in the absence of major diseases. PA influences a person’s physical and psychological health and functional status as well as the self-perception of “aging well”, maintaining a good quality of life [5].

The age-related progressive deterioration in skeletal muscle mass, strength, and physical function is known as sarcopenia [6,7]. Sarcopenia is associated with multiple adverse health events, including cardiovascular problems, functional disability, and increased fall incidence, hospital admissions, and mortality [8,9,10,11,12,13]. Subjects with sarcopenia have demonstrated a significantly high proportion of problems related to several dimensions of quality of life. 

More than 10% of individuals aged 60–69 years and approximately 40% of adults over 80 years of age are affected by sarcopenia [14,15]. The loss of autonomy and the increasing risk of additional diseases caused by sarcopenia represents a significant problem also for public health systems, which, as longevity increases, is projected to increase in the next decades [16]. 

While skeletal muscle properties are known to be highly heritable, evidence regarding the specific genes related to muscle strength and aging is currently inconclusive. 

In the past 20 years, attention has been paid to the identification of specific genes and single-nucleotide polymorphisms (SNP) in elite athletes, attributing heritable characteristics to muscle strength and physical state [17,18,19,20]. One of the most studied genetic polymorphism is *ACTN3* NM_001104.4 (ACTN3_v001):c.1729C>T at exon 15 or *ACTN3* [rs1815739] where arginine (R) becomes a stop (X) codon at position 577 (R577X) [21]. The protein α-actinin-3 encoded by the *ACTN3* gene is one of the main structural components of the muscle fiber Z disc, which can anchor actin filaments in the sarcomere [22] and bind to a variety of structural, metabolic, and signaling proteins [23]. The main function of α-actinin-3 seems to be structural. This protein is only expressed in type II muscle fibers. Therefore, individuals with the *ACTN3* 577XX genotype are deficient in α-actinin-3 protein, which is associated with a lower fast-twitch fiber percentage [24], and cannot produce α-actinin-3 protein in muscle. It is estimated that the incidence of this genetic variation is 16–18% in the total population [25,26]. 

At first, α-actinin-3 deficiency in the general population seemed to be related to the decline of physical strength with age [27]. The relationship between strength and muscle mass in elderly people was studied to reduce mortality [28], and the influence of the *ACTN3* gene R577X polymorphism on muscle phenotype and bone mineral density in this population is not well established. In addition, the role of this polymorphism in health-related quality of life (HRQoL) or morbidity in this population is of great interest.

Knowledge of individual *ACTN3* genotypes could provide valuable information for the management of risk factors in the elderly and promote preventive measures aimed at improving quality of life during ageing through the personalization of preventive interventions [29].

## 2. Material and Methods

### 2.1. Participants

Two hundred and eighty-one older adults (over 65 years old) were recruited for the study. The population was selected by simple random sampling at different primary-care centers. All recruited participants were Caucasian descendents from three or more generations. Written, signed informed consent was obtained from all subjects. The inclusion criteria were adults over 65 years old, not institutionalized, and not affected by dementia or mobility impairments. The study was conducted according to the guidelines of the Declaration of Helsinki and approved by the Area de Salud de Burgos y Soria Ethics Committee (Ref. CEIC 1446).

Data were collected by a research nurse, and the following socio-demographic characteristics were included: body mass index (BMI), age, gender (male, female), prescribed drugs, falls and hospital admissions during the last year, and clinical group risk (CGR) category. The CRGs category is defined using a claims-based classification system for risk adjustment that assigns each individual to a single risk group (among mutually exclusive ones) based on historical clinical (morbidity and chronicity) and demographic characteristics, to predict the future use of healthcare resources [30].

### 2.2. Assessment of Quality of Life, Physical Activity, and Nutritional Status

To evaluate quality of life, the EuroQoL five-dimension questionnaire (EQ-5D) was used. This questionnaire, which has been validated in Spanish [31,32,33], allows a standardized measure of HR-QoL and the use of a EQ-5D visual analogue scale (EQ-VAS), that can be applied for a wide range of health conditions and treatments. This descriptive system evaluates the patient state of health in five dimensions: mobility, self-care, usual activities, pain/discomfort, and anxiety/depression. Each dimension has three levels: no problems, some problems, and severe problems, and the patient has to evaluate each dimension. The results are combined in a unique parameter corresponding to the participant’s health state and then a final EQ-5D index is calculated. The EQ-VAS records an individual’s self-rated health on a vertical visual analogue scale. This is used as a quantitative measure of health outcome that reflects the subjects’ own judgement. The EQ-VAS measures the patient’s self-rated health on a 20 cm vertical, visual analogue scale with endpoints labelled as “the best health you can imagine” and “the worst health you can imagine” [34]. The advantages of this questionnaire are that it is short, easy to complete, and simple to understand [33].

For the analysis of physical activity levels, the participants completed the questionnaire called Physical Activity Scale for the Elderly (PASE), which is a validated 12-item questionnaire that is designed to measure the level of physical activity in individuals over the age of 65.

The PASE questionnaire assesses basic activities of older adults (walking, recreational activities, exercise, housework, yard work, and caring for others). It recovers frequency, duration, and intensity level of the activities over the previous week to assign a score ranging from 0 to 793, with higher scores indicating greater levels of physical activity [35].

To assess the nutritional status, the Mini Nutritional Assessment (MNA) questionnaire was used. Older adults were classified as well nourished, at risk for malnutrition, or malnourished. The MNA has 18 questions for the evaluation anthropometric, general, dietary, and self-care parameters. We performed the full MNA for all subjects [36].

### 2.3. Physical Performance Measures

Muscular strength was assessed using the hand grip strength (HGS) test. HGS was measured in the dominant hand (the average score of three measures was used in the analyses) by a maximal isometric test using a hand dynamometer [37]. Analyses of HGS were undertaken by age and gender. The European Working Group on Sarcopenia in Older Persons defined weakness on the basis of a HGS less than 30 kg in men and less than 20 kg in women [38]. For the identification of participants with clinically meaningful weakness, HGS was classified in two categories as follows: weak-intermediate and normal, according to cut-off values published by Alley et al., 2014 [39].

### 2.4. Genotyping

Genomic DNA was purified from total blood using a specific extraction kit (G-spin™ Total DNA Extraction Mini Kit, Intronbio, Seongnam, Korea), and genotyping analyses were performed in the Genetics Laboratory of the Universidad de Valladolid (Soria, Spain). Our study followed recent recommendations for replicating genotype–phenotype association studies [40]: genotyping was performed only for research purposes, and the researchers responsible of genotyping were totally blinded to personal identities.

For *ACTN3* R577X genotyping, we used real-time PCR and Taqman probes with a Step One Real-Time PCR System (Applied Biosystems, Foster City, CA, USA).

### 2.5. Statistical Analysis

The main characteristics are presented as the mean ± standard deviation (SD) or as a percentage. Student’s *t*-test or analysis of variance (ANOVA) was used for continuous variables, and the Chi-square test was used for categorical variables. Deviation from Hardy–Weinberg equilibrium for the *ACTN3* R577X polymorphism was tested by the chi-squared test. Values were considered statistically significant when *p* < 0.05, and all the *p*-values were two-sided.

Interaction analysis between the R577X genotypes and age and gender in relation to physical performance, QoL, or chronicity phenotypes was conducted using a general linear model and further by stratification analysis. To estimate the associations of the genetic variants with physical phenotypes, a regression coefficient was derived from linear regression models in SPSS, version 19.0 (SPSS Inc., Chicago, IL, USA).

## 3. Results

We analyzed a total of 281 subjects without missing values; 46.6% of them were men, and 53.4% were women, and their age ranged from 64 to 94 years, with an average of 76.1 (± 7.1).

The characteristics of the participants divided by gender are shown in Table 1. Significant differences were observed between men and women in number of falls and HGS values (*p* < 0.001 and *p* < 0.001, respectively). Males presented greater HGS values than females, and females reported more falls during the last year. The mean values of HGS were 45.6 kg for men and 30.7 kg for women. For men, a grip strength less than 32 kg was classified as “intermediate-weak”; 33.6% of men were intermediate-weak. For women, a grip strength less than 20 kg was classified as “intermediate-weak”; 24% of women were intermediate-weak (data not shown).

The average PASE score suggested no differences between males and females (Table 1). The MNA score suggested that 89.4% of the participants had a normal nutritional status and 10.6% were at risk for malnutrition, with no significant sex differences.

As expected, for both men and women, after Spearman correlation analysis, we observed a negative correlation between age and HGS (*p* < 0.001 and *p* < 0.001, respectively) and a positive correlation with the number of prescribed drugs (*p* < 0.001 and *p* = 0.004, respectively). In addition, for both groups, the PASE value was negatively correlated to EQ-VAS (*p* = 0.018 and *p* < 0.001, respectively) and EQ-5D (*p* < 0.001 and *p* < 0.001, respectively). For men, we also observed a marginal negative correlation between number of prescribed drugs and PASE value (*p* = 0.016), as shown in Table 2 and Table 3.

For the selected population, the genotype distribution of the *ACTN3 R557X* polymorphisms was 31.5% RR genotype, 48.5% RX genotype, and 20% XX genotype. The genotype frequencies were in Hardy–Weinberg equilibrium (*p* = 0.865).

As shown in Table 4, multivariate regression analysis revealed in adjusted model that in men, the *ACTN3 R577X* genotype was significantly associated with HGS, regression coefficient (β) = 1.23, *p* = 0.008; dimension 1 of EQ-5D (mobility), (β) = −1.44, *p* = 0.006, and CGR category (β) = −1.38, *p* = 0.006. In women, a marginal association between the *ACTN3 R577X* genotype and CGR category was observed, with a regression coefficient of (β) = −0.97, (*p* = 0.024) (Table 4). However, in women, no significant association was observed between the *ACTN3 R577X* genotype and HGS or mobility (see Table 4).

In addition, in both men and women no significant association was observed between the *ACTN3 R577X* genotype and the remaining dimensions of EQ-5D and EQ-VAS (Table 4).

## 4. Discussion

There is not much information available regarding the health conditions (EQ-5D, EQ-VAS, HGS, chronicity, and use of medications, among others) and the physical activity levels in older adults (65 or older).

The process of aging increases the risk of a number of diseases. Some studies suggest that increasing the levels of PA in the elderly population could not only postpone the development of chronic diseases but also optimize healthcare systems [41,42]. Drugs consumption, in general, can be an indicator of the overall health status, and some studies show that individuals who practice low levels of PA tend to consume more medications or more healthcare resources, as compared to individuals with higher levels of PA [43]. We also observed a negative correlation between number of prescribed drugs and PASE, EQ-VAS, and EQ-5D.

In addition, PA can benefit the QoL and also shows a positive impact on depression. Our results agree with previous published studies, showing that participants with higher levels of PA presented a higher EQ-5D index and self-rated health outcome (EQ-VAS) [44,45,46].

This study, to our knowledge, is the first to examine the relationship between QoL (EQ-5D), muscular strength (HGS), and chronicity/morbidity (CGR category) and the *ACTN3 R577X* polymorphism in an older Spanish population.

In our study, men older than 65 years of age showed differences in HGS, mobility (dimension 1 of EQ-5D), and CGR category according to the *ACTN3 R577X* genotype. On the other hand, for women we only found a statistically significant association between CGR category and *ACTN3 R577X* genotype.

In man, we observed that the *ACTN3 577XX* genotype was associated with higher HGS values, not having any problem in mobility, and being in group 0 or 1 of CGR category (*p* = 0.006, *p* = 0.008 and *p* = 0.006, respectively). For women, the *ACTN3 577XX* genotype was marginally associated with being in CRG group 0 or 1 (*p* = 0.024). Recently, Ma et al. found evidence of gender- and age-specific associations of *ACTN3 R577X* genotypes with physical performance phenotypes (including HGS) in older populations [47]. In addition, Dato and colleagues reported that the genetic component of frailty was higher among males than among females and higher in older subjects [48].

Physical performance measures including HGS are associated with healthy aging, and lower scores increase the risk of mortality [49,50]. Muscle strength and mass are protective against all causes of mortality in elderly [28].

Previous studies have shown that the *ACTN3* genotype is a modulator of muscle mass and function and of sarcopenia risk in elderly adults, initially being the allele R of *ACTN3 R577X* associated with greater maintenance of strength and function or with sarcopenia protection [24,51,52]. Better strength associated with allele R has been frequently studied in athletes [53]. From a physiological point of view, this relationship could be due to the association of the R allele with an increase in type II muscle fibers and the ability to maintain fast-twitch fiber size and mass with age in these subjects [24]. Surprisingly, in elderly people, some authors have reported that better results in strength tests were associated with the *ACTN3 XX* genotype, while for other researchers this genotype appeared as the least favorable [54]. 

Lifestyle habits of the elderly, such as regularly practicing physical exercise, seem to be determinant. Recently, Romero-Blanco published that women with the *ACTN3 XX* genotype improved their muscle strength after 24 months of training (in the study they tried to homogenize the characteristics of the participants, such as training, gender, age and lifestyle) [55]. Seto et al. hypothesized that the absence of α-actinin-3 produces an increase of calcineurin activity, which reprograms the metabolic phenotype of fast muscle fibers and results in better adaptation of skeletal muscles to training [56]. Moreover, Garton et al. suggested that α-actinin-3 deficiency also protects against muscle wasting [57].

While the deficiency of α-actinin-3 has no apparent association with muscle diseases, there is an established relationship with morbidity in people who are frail, suggesting that in centenarians, it could provide a survival advantage [58].

Deschamps et al. reported that centenarians with the *ACTN3* XX genotype may be less predisposed to chronic diseases [59]. We have also observed an association between the *ACTN3* XX genotype and being in the G0 or G1 CGR (healthy or with an acute process (G0) and single minor chronic disease (G1)). While several methods are available to identify frail patients, there are no guidelines for the identification of complex elderly patients, who often present high levels of multi-morbidity. Yet, it is well known that multi-morbidity in the elderly is associated with poor outcomes, and the commonly used CGR classification system is a good tool to evaluate this situation.

On the other hand, as far as we know, there are no published studies evaluating the relationship between QoL and the *ACTN3* genotype. We found a statistically significant association between dimension 1 of EQ-5D and the *ACTN3* R577X polymorphism. Participants with the XX genotype had a higher probability of not having any problem in mobility than those without this genotype. These results are also in accordance with the association of the *ACTN3 XX* genotype with HGS values that we observed. Sarcopenia is associated with healthy outcomes and an obvious decline in QoL.

Seto et al. [60] reported that genotype differences in fast muscle force production result in fast-twitch fibers developing slower activities, suggesting that the lack of alpha-actinin-3 may cause a faster decrease in muscle function with increasing age. The loss of type II muscle fibers may be particularly important concerning the influence of the *ACTN3 R577X* in the elderly, as *ACTN3* is mainly expressed in this fiber type. Moreover, people without α-actinin-3 show better adaptation to resistance training [61].

This study has several limitations. For example, muscle strength is a complex phenotype, which is likely influenced by numerous genes and genetic variants, as well as other environmental factors that may be interacting with these genes in several pathways. The sample group was divided by gender, which may have reduced the statistical power. On the other hand, despite the small sample size of the current study, our population was homogeneous and well defined in terms of phenotype assessment, and it is known that sarcopenia may be gender-dependent. To our knowledge, this is the first study to evaluate the relationship between QoL and the ACTN3 genotype. Moreover, the study follows the STREGA guidelines, all participants were randomly recruited by a research nurse, genetic polymorphism was selected considering its prevalence and functional impact, no departure from Hardy-Weinberg equilibrium was detected, nor multiple testing and reporting of quantitative (continuous) outcomes were used. A better reporting in studies facilitates the synthesis of research results and the further development of study methods in genetic epidemiology improving the understanding of the role of genetic factors.

## 5. Conclusions

In this study, we found evidence of gender-specific associations of the ACTN3 R577X polymorphism with muscular strength, QoL, and morbidity in the older population. Our results support the hypothesis that the lack of alpha-actinin-3 may cause a faster decrease in muscle function with increasing age. Nevertheless, the specific underlying mechanisms will require further investigation. Establishing the influence of the ACTN3 R577X variant on functional health status or on quality of life in older adults is necessary to determine if this genotype could be useful for identifying individuals who may be more susceptible to sarcopenia and who may need specific global health interventions.

### Key Points

We found an association between the ACTN3 R577X genotype and muscular strength in older men.

We found an association between the ACTN3 R577X genotype and the dimension of mobility of EQ-5D in older men.

Our results support the hypothesis that the lack of alpha-actinin-3 may cause a faster decrease in muscle function with increasing age.

We found an association between the ACTN3 R577X genotype and chronicity and multimorbidity in Spanish older adults.

## Figures and Tables

**Table 1:** Characteristics of the Study Participants (N =281).

Characteristics	Men *n* = 131	Women *n* = 150	*p*-Value
Age, mean (SD)	76.69 (7.32)	75.68 (6.92)	0.240
BMI, mean (SD)	27.59 (3.82)	27.68 (4.20)	0.842
Total drugs, mean (SD)	5.00 (3.53)	4.51 (3.38)	0.245
Falls, mean (SD)	0.07 (0.26)	0.26 (0.51)	<**0.001**
Hospital admissions, mean (SD)	0.10 (0.30)	0.09 (0.29)	0.747
CRG (% pluripathologic or chronic diseases)	34.10	26.70	0.111
EQ-5D, mean (SD)	0.83 (0.16)	0.88 (1.07)	0.617
EQ-VAS, mean (SD)	73.58 (13.82)	69.66 (17.36)	0.039
PASE score, mean (SD)	269.07 (169.84)	254.38 (158.38)	0.454
HGS, mean (SD)	45.63 (25.80)	30.76 (16.10)	<**0.001**
MNA, mean (SD)	27.04 (2.65)	26.78 (2.92)	0.440

Note: Values are percentages for categorical data and mean and standard deviation for continuous data. SD, standard deviation; BMI, body mass index; CRG, Clinical Risk Groups; EQ-5D, EuroQol 5-Dimension questionnaire; EQ-VAS, EuroQol Visual Analogue Scale; VAS, Visual Analogue Scale; PASE, Physical Activity Scale for the Elderly; HGS, Hand Grip Strength; MNA, Mini Nutritional Assessment. Statistically significant variables are in bold.

**Table 2:** Pearson correlation coefficients of the analyzed variables in men.

	Age	BMI	Drugs	Falls	Hospital Admissions	PASE Score	HGS	EQ-VAS	EQ-5D
**Age**	1	−0.118	0.371	0.045	−0.014	−0.036	−0.312	−0.159	−0.203
		0.180	**<0.001**	0.611	0.874	0.686	**<0.001**	0.070	**0.020**
**BMI**		1	0.150	0.030	−0.041	−0.092	0.073	−0.107	−0.164
			0.096	0.763	0.653	0.297	0.406	0.225	0.061
**Drugs**			1	0.089	0.152	−0.216	−0.118	−0.283	−0.184
				0.334	0.100	**0.016**	0.191	**<0.001**	0.041
**Falls**				1	0.125	−0.013	0.090	−0.036	0.053
					0.165	0.881	0.314	0.690	0.549
**Hospital admissions**					1	0.080	0.089	−0.163	−0.094
						0.377	0.325	0.070	0.300
**PASE score**						1	−0.077	0.206	0.353
							0.382	**0.018**	**<0.001**
**HGS**							1	0.099	0.052
								0.258	0.556
**EQ-VAS**								1	0.291
									**0.001**
**EQ-5D**									1
									-

Note: Each cell contains two values: (a) Pearson correlation coefficient; (b) *p* value, indicating if the correlation is significant. Statistically significant variables are in bold.

**Table 3:** Pearson correlation coefficients of the analyzed variables in women.

	Age	BMI	Drugs	Falls	Hospital Admissions	PASE Score	HGS	EQ-VAS	EQ-5D
**Age**	1	0.025	0.240	0.028	0.012	0.000	−0.293	−0.071	−0.136
		0.759	**0.004**	0.741	0.892	0.999	**<0.001**	0.387	0.098
**BMI**		1	0.137	−0.022	−0.063	−0.033	0.092	−0.010	−0.085
			0.101	0.759	0.455	0.685	0.262	0.902	0.304
**Drugs**			1	−0.065	0.042	−0.128	−0.086	−0.328	−0.382
				0.455	0.626	0.126	0.303	**<0.001**	**<0.001**
**Falls**				1	0.177	−0.135	−0.034	−0.094	−0.006
					**0.036**	0.110	0.685	0.268	0.944
**Hospital admissions**					1	−0.013	0.061	0.045	−0.022
						0.883	0.469	0.593	0.799
**PASE score**						1	−0.121	0.481	0.391
							0.142	**<0.001**	**<0.001**
**HGS**							1	0.188	0.081
								**0.021**	0.326
**EQ-VAS**								1	0.507
									**<0.001**
**EQ-5D**									1
									-

Note: Each cell contains two values: (a) Pearson correlation coefficient; (b) *p* value, indicating if the correlation is significant. Statistically significant variables are in bold.

**Table 4:** Logistic regression of the association between *ACTN3 R577X* polymorphism (recessive model XX/(RR+RX) (rf)) and each dimension of EQ-5D, EQ-VAS, HGS, and CRG.

	Men				Women			
	Crude Model Β (SE)	*p* Value	Adjusted Model Β (SE)	*p* Value	Crude Model Β (SE)	*p* Value	Adjusted Model Β (SE)	*p* Value
EQ-5D *								
Mobility	0.59 (0.50)	0.238	−1.44 (0.52)	**0.006**	0.63 (0.66)	0.339	−0.25 (0.40)	0.529
Self-care	−0.57 (1.25)	0.645	−0.505 (1.43)	0.604	1.00 (1.12)	0.368	−0.63 (0.59)	0.284
Usual-activities	−0.42 (0.77)	0.591	−1.66 (1.17)	0.157	−0.04 (0.76)	0.958	−0.31 (0.53)	0.553
Pain and discomfort	0.59 (0.50)	0.238	0.082 (0.446)	0.854	−0.01 (0.58)	0.984	−0.44 (0.38)	0.253
Depression and anxiety	0.34 (0.54)	0.531	−0.32 (0.49)	0.523	0.92 (0.62)	0.134	−0.63 (0.38)	0.100
**EQ-VAS**	−0.42 (0.72)	0.561	0.26 (0.618)	0.996	−0.59 (0.85)	0.482	−0.046 (0.48)	0.924
**HGS**	−1.08 (0.50)	**0.031**	1.23 (0.47)	**0.008**	0.01 (0.63)	0.991	0.42 (0.43)	0.337
**GCR**	0.13 (0.48)	0.777	−1.38 (0.51)	**0.006**	0.05 (0.60)	0.962	−0.97 (0.43)	**0.024**

* Problems in each dimension of EQ-5D. Statistically significant variables are in bold.

## Data Availability

Data sharing not applicable.
